# Clinical Application Value of Circulating Cell-free DNA in Hepatocellular Carcinoma

**DOI:** 10.3389/fmolb.2021.736330

**Published:** 2021-09-29

**Authors:** Yuyuan Zhang, Zaoqu Liu, Kun Ji, Xin Li, Caihong Wang, Zhigang Ren, Yang Liu, Xinju Chen, Xinwei Han, Lingfang Meng, Lifeng Li, Zhen Li

**Affiliations:** ^1^ Department of Interventional Radiology, The First Affiliated Hospital of Zhengzhou University, Zhengzhou, China; ^2^ Interventional Institute of Zhengzhou University, Zhengzhou, China; ^3^ Interventional Treatment and Clinical Research Center of Henan Province, Zhengzhou, China; ^4^ Department of Magnetic Resonance, The First Affiliated Hospital, Zhengzhou University, Zhengzhou, China; ^5^ Department of Infections Disease, The First Affiliated Hospital, Zhengzhou University, Zhengzhou, China; ^6^ Department of Radiation Oncology, The Affiliated Cancer Hospital of Zhengzhou University, Zhengzhou, China; ^7^ First Ward of Spleen, Stomach, Liver and Gall, The First Affiliated Hospital of Henan University of TCM, Zhengzhou, China; ^8^ Department of Ultrasound, Zhengzhou Sixth People’s Hospital, Henan Infectious Disease Hospital, Zhengzhou, China; ^9^ Internet Medical and System Applications of National Engineering Laboratory, Zhengzhou, China; ^10^ Cancer Center, The First Affiliated Hospital of Zhengzhou University, Zhengzhou, China

**Keywords:** cfDNA, CtDNA, Hepatocellular carcinoma, clinical application, biomarkers

## Abstract

Hepatocellular carcinoma (HCC) is one of the most common cancers worldwide and a leading cause of cancer-related deaths. Due to late diagnosis, early intrahepatic metastasis and nonresponse to systemic treatments, surgical resection and/or biopsy specimens remain the gold standard for disease staging, grading and clinical decision-making. Since only a small amount of tissue was obtained in a needle biopsy, the conventional tissue biopsy is unable to represent tumor heterogeneity in HCC. For this reason, it is imperative to find a new non-invasive and easily available diagnostic tool to detect HCC at an early stage and to monitor HCC recurrence. The past decade has witnessed considerable evolution in the development of liquid biopsy technologies with the emergence of next-generation sequencing. As a liquid biopsy approach, molecular analysis of cell-free DNA (cfDNA), characterized by noninvasiveness and real-time analysis, may accurately represent the tumor burden and comprehensively reflect genetic profile of HCC. Therefore, cfDNA may be used clinically as a predictive biomarker in early diagnosis, outcome assessment, and even molecular typing. In this review, we provide an update on the recent advances made in clinical applications of cfDNA in HCC.

## Introduction

Hepatocellular carcinoma (HCC) is the sixth most common cancer worldwide and the fourth most frequently reported cause of cancer death by 2018 (Bray et al., 2018). HCC ranks third of cancer-related mortality in China, and the major risk factors for HCC are hepatitis B virus (HBV) or hepatitis C virus (HCV) infection, exposure to aflatoxin B1, alcohol consumption, and metabolic disorders (Chen et al., 2016). Currently, benefiting from liver resection, ablation, liver transplantation, the 5-years survival rate of early HCC (BCLC stage A) can reach 50–75%. Unfortunately, owing to the paucity of specific symptoms and early intra/extrahepatic metastases, most patients have already reached an advanced cancer stage at the time of first HCC diagnosis, giving rise to fewer than 40% of HCC patients eligible for surgical intervention (Forner et al., 2018). Therefore, it is critical to find a robust method to detect patients with HCC at earlier stages, monitor tumor recurrence and even better predict response to treatment in a dynamic and real-time manner.

The diagnosis and surveillance of HCC primarily depends on findings from imaging analysis and alpha-fetoprotein (AFP) levels. Unfortunately, this dynamic imaging has limitations in accuracy and sensitivity when referring to small or hypovascularized lesions. Biopsy is recognized as the standard diagnosis method, while the problem of invasiveness and the false positivity remains to be solved. Lately, circulating free DNA (cfDNA) has emerged as a promising alternative in tumor diagnosis, recurrence surveillance and druggable targets identification (Benesova et al., 2013; Marzese et al., 2013; Diaz and Bardelli, 2014). CfDNA is the fragmented DNA in the blood circulation which can be detected in healthy individuals and patients with cancer, and ctDNA is the fraction of cfDNA specifically derived from primary or metastatic tumors, with the concentration ranging from 0.01 to 90% (Jen et al., 2000). Nowadays, both qualitative and quantitative analysis of cfDNA is utilized in cancer based on underlying genetic predisposition, including detection of genomic changes, mutational analysis, oncogenic pathway determination, prediction/monitoring of treatment response, drug resistance alterations, and identification of mechanisms of malignant/metastatic transformation (Kaseb et al., 2019).

HCC is a highly heterogeneous disease attributing to the accumulation of somatic genomic aberrations in passenger and driver genes as well as epigenetic modifications (Forner et al., 2018). For many years, the recognition of genomic aberrations in HCC mainly relied on liver resection/biopsies. Since invasiveness, traditional fine-needle biopsy does not entirely avoid risks and potential complications, such as pain (84%) (Eisenberg et al., 2003), bleeding (Russo et al., 2018) and needle tract seeding (2.7%) (Silva et al., 2008). In addition, insufficient material for clinical sequencing occurs in 20–25% of needle biopsies (Zill et al., 2015), resulting in the mental and financial pressure of patients to some extent. Intrahepatic metastasis occurs early in the progression of HCC and thus information acquired from a needle biopsy of a single tumor lesion might fail to reflect the tumor burden (Swanton, 2012). In turn, analysis of cfDNA may overcome these limitations and subsequently provide the genomic profiles of all lesions (both primary lesion and metastasis), and this method can be utilized to track genomic evolution systematically and dynamically (Kaseb et al., 2019). With increasing availability and reliability in high-throughput technology, plasma cfDNA may meet the demands of disease surveillance, management of different stages and personal precision medicine for patients with HCC. This review will provide an update on the advances made in the clinical application of cfDNA in HCC in recent years.

### cfDNA

#### Biological Basis

cfDNA is double-stranded DNA measuring approximately 150–200 base pairs which exists in the plasma or serum (Fan et al., 2010). Typically, the concentration of cfDNA is low (10–15 ng/ ml on average) for healthy people, with a short half-life of between 16 min and 2.5 h (Lo et al., 1999; Diehl et al., 2008). While, increasing concentration of cfDNA can be observed under some physical and pathological circumstances, such as exercise, inflammation, surgery, autoimmune disease, and transplantation (Diehl et al., 2008). These characteristics indicate that cfDNA can provide more real-time information regarding the cancer status than routine serum biomarkers, such as AFP, CEA and CA-199.

**FIGURE 1:**
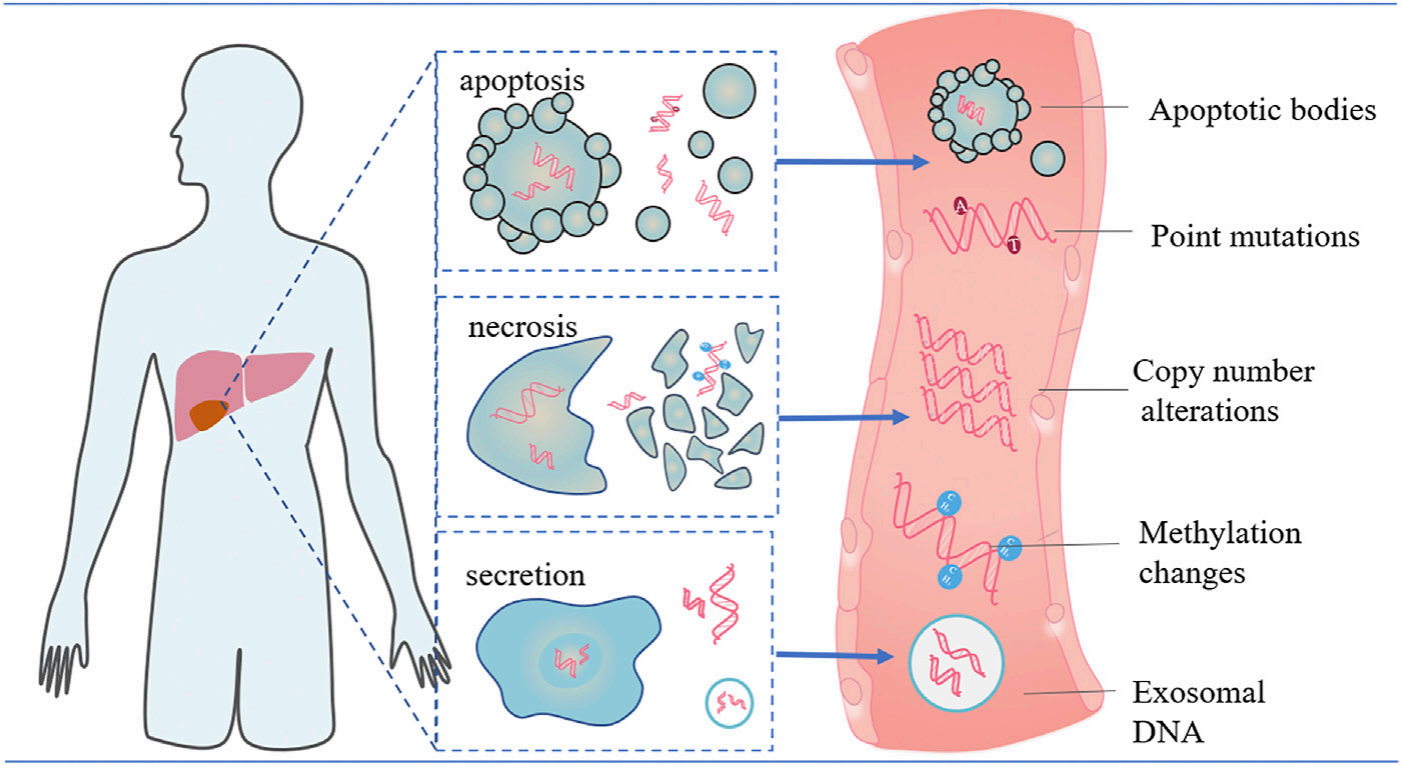
Origin and range alterations of cfDNA. Cell-free DNA (cfDNA) is passively released by apoptotic or necrotic tumor cells and actively secreted by exosomes, which are membrane -bound vesicles released by tumor cells. Of these cells, apoptosis serves as the main source of cfDNA in both normal and diseased tissues. Analysis of these molecules can be employed for early tumor detection and may provide a prognostic treatment strategy for HCC patients.

Although the clinical benefits of cfDNA have been increasingly recognized recently, many aspects of the biological characteristics of tumor-derived cfDNA still have not been elucidated clearly. Firstly, the origin of cfDNA has not been definitively clarified with several possible mechanisms proposed (Figure 1). Two possibilities exist for the main origins of cfDNA: cellular breakdown mechanisms (such as apoptosis and necrosis) and active DNA release mechanisms (such as exosomes, virtosomes and argonaute) (Aucamp et al., 2018). Tumor-associated DNA is primarily derived from apoptosis of primary, metastatic and circulating tumor cells (CTCs), as well as active release from proliferative tumor cells, which is known as circulating tumor DNA (ctDNA) (Crowley et al., 2013). In general, ctDNA only accounts for a small fraction of the total cfDNA and there is no way to isolate ctDNA, especially from other cfDNA. Only the emergence of tumor-related molecular alterations in cfDNA indicates the presence of ctDNA (Ye et al., 2019).

In addition, there are still some different views concerning circulating DNA fragment patterns derived from tumors such as the concentration of total ctDNA and the proportion of tumor-derived ctDNA fragments (Umetani et al., 2006a; Gao et al., 2010; Mouliere et al., 2011). A number of studies reported the finding of increased integrity of tumor-derived plasma DNA (Wang et al., 2003; Jiang et al., 2006). For example, a study reported that the increased DNA integrity in plasma DNA is associated with cancer, and excellent performance was achieved in detecting the cancer group from the nonneoplastic group. On the other hand, there is also seemingly contradictory evidence that plasma DNA molecules released by tumors might be shorter (Diehl et al., 2005; Mouliere et al., 2011). For example, a study performed by massively parallel sequencing suggested that the size of plasma DNA molecules harboring tumor-associated genomic alterations was shorter (Jiang et al., 2015). Overall, cfDNA in the plasma is vulnerable to various assay platforms and physiological state, and thus it is necessary to comprehensively analyze tumor-derived plasma DNA rather than treating without distinction.

#### Technology Platform for ctDNA

**FIGURE 2:**
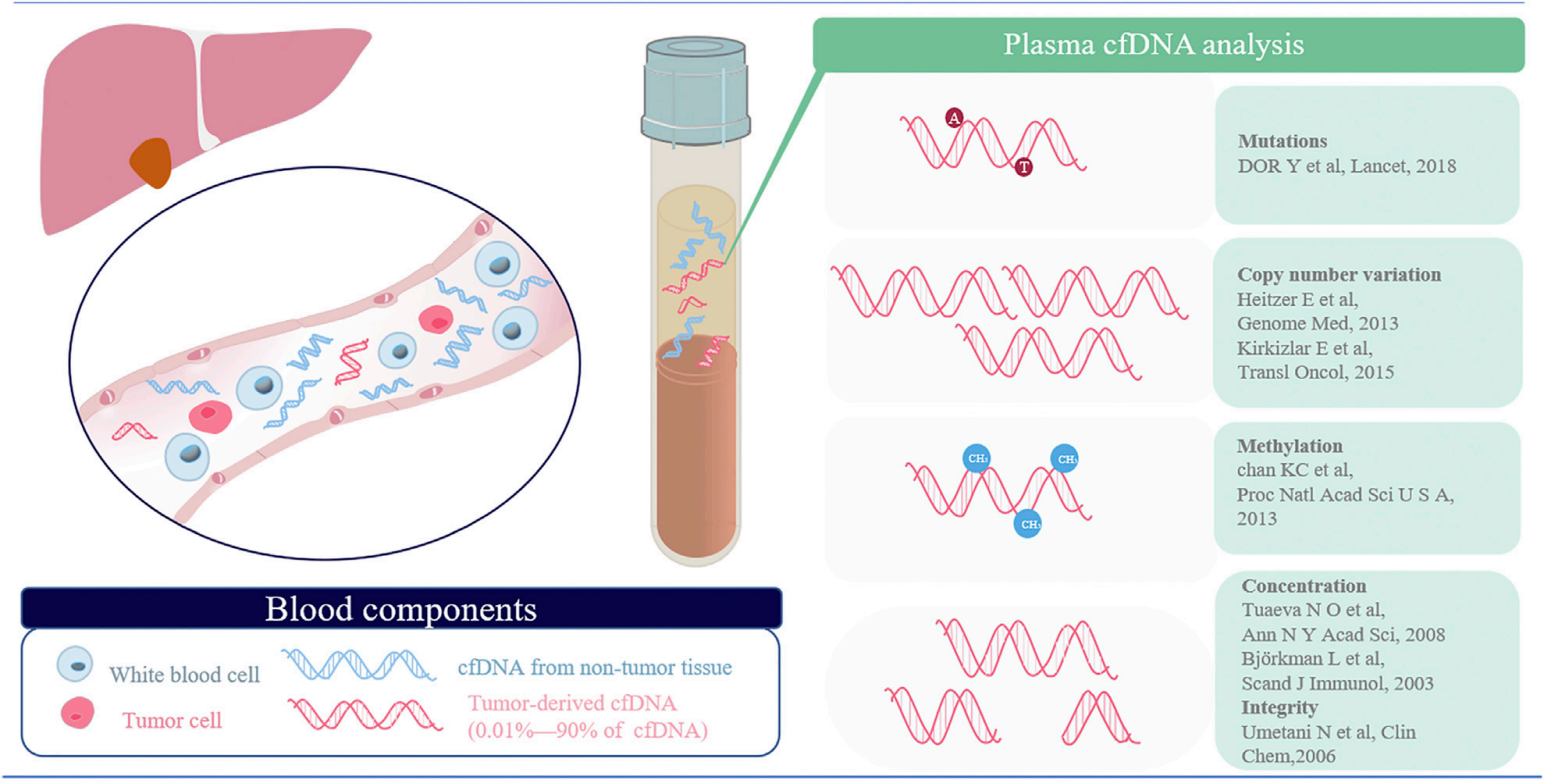
Overview of the methods employed in the detection of different cancer-associated alterations. cfDNA in the plasma captures different cancer-associated changes, including mutations, copy number aberrations, alterations in DNA methylation and altered DNA fragmentation patterns (concentration and integrity).

CtDNA accounts for only a small percentage of the total cfDNA in the peripheral blood and is highly variable from patient to patient (0.01–90%), which therefore necessitates the utilization of hypersensitive and highly specific approaches (Corcoran and Chabner, 2018). Typically, alterations of cfDNA in patients with cancers involve quantity and quality (Gao et al., 2017). The former refers to the total concentration and integrity, and the latter means genetic or epigenetic alterations containing single nucleotide mutations, copy number variations and methylation changes and so on. In summary, the techniques for the analysis of cfDNA can be summarized as targeted methods, such as digital PCR, BEAMing digital PCR, and amplification-refractory mutation system (ARMS)-PCR, as well as untargeted methods, such as whole-genome sequencing (WGS) and next-generation sequencing (NGS). Although the former method can screen cfDNA aberrations dynamically and has a very high sensitivity, it detects mutations only in a set of predefined genes based on prior knowledge (Oh et al., 2010). A more comprehensive view of the entire genomic landscape may be exhibited using the latter method, which may provide the potential of identifying drug resistance genes and recognizing novel actionable targets (Slatko et al., 2018). In general, the rational combination of the two techniques would accomplish the purpose, providing a noninvasive approach to guide the clinical management of patients. In the following part, we will detailedly introduce the techniques for detecting aberrant cfDNA in the plasma (Figure 2).

#### Concentrations

In 1977, for the first time, researchers revealed that cfDNA increased in patients with tumors (Leon et al., 1977). After this seminal work, a significant volume of researches have attempted to detect cancers by quantifying the absolute concentration of cfDNA in the serum/plasma (Mehra et al., 2018; Valpione et al., 2018; Wu et al., 2019). At present, the level of cfDNA in the plasma was estimated by several classical methods, including fluorescence-based quantitative real-time PCR (qPCR), histochemical techniques (such as Hoechst staining and PicoGreen staining) and optical imaging techniques (such as ultraviolet measurement) (Björkman et al., 2003; Tuaeva et al., 2008). It appears that the level of cfDNA in the plasma may reflect the tumor burden (Yang et al., 2011) and may have diagnostic value for HCC (Huang et al., 2012; Yan et al., 2018; Qu et al., 2019). However, an increase in the concentration alone cannot be regarded as an effective and cancer-specific metric for diagnosis and classification because the cfDNA in the plasma is vulnerable to various assay platforms and physiological state like active ongoing hepatitis and hepatic dysfunction (Yan et al., 2018).

#### Integrity

There is another quantitative characterization of tumors associated with cfDNA: assessment of the integrity index. Currently, the most popular method was published by Umetani. By developed the qPCR method for ALU repeats, this method accomplished the assessment of the integrity without DNA purification and then established the integrity index (Umetani et al., 2006b). In the ALU-qPCR, DNA integrity was determined as the ratio of ALU247 primers to ALU115 primers, and the ALU115 primers represent the absolute amount of DNA. Plasma cfDNA integrity has been applied to many types of cancers, such as breast cancer (Umetani et al., 2006a), colon cancer (Agostini et al., 2011) and liver cancer (Huang et al., 2016a). Compared with concentrations of cfDNA in the plasma, cfDNA integrity is less affected by much influential factors. In previous studies (El-Shazly et al., 2010; Chen et al., 2012; Huang et al., 2016a), changes of integrity in cfDNA is associated with large tumor size and vascular invasion, and it exerted a crucial role in distinguishing cancer from healthy people and surveillance of residual disease after surgery.

#### Mutations

Studies identifying and characterizing somatic genetic mutations in HCC have been updated annually. There are two types of methods for detecting mutation: targeted methods monitoring interesting gene loci and nontargeted massively parallel sequencing. When the proportion of cfDNA derived from tumor cells accounts for only a small part of the cfDNA in the plasma, abundant errors are expected to be present in the PCR amplification and sequencing procedures. To overcome this problem, unique molecule identifiers (UMIs) have emerged for detecting single nucleotide mutations at a fraction as low as 1 in 1 million molecules (Kinde et al., 2011). In the clinical practice, compared with tumor tissue level mutational heterogeneity, single specific ctDNA alterations do not have sufficiently high sensitivity or specificity as a diagnostic biomarker for HCC. In past literatures, a positive rate of 20–56% for plasma ctDNA was reported (Huang et al., 2016b; Jiao et al., 2018a). Improvement in NGS technology allowed comprehensive analysis of the mutational landscape of ctDNA, expanded the list of mutation biomarker candidates, and promisingly improved the detection rate of tumor-related mutation with the positive rate of 60–80% (Ng et al., 2018; Yang et al., 2019).

#### Methylation

Epigenetic events play a crucial role in hepatocarcinogenesis. Methylation is a covalent modification pattern that can exist stably in the plasma after the release of defective cells (Dor and Cedar, 2018). Currently, the detection of methylation primarily consists of two types based on the size of the targeted region: target-region methylation detection (methylation-specific PCR) and genome-wide methylation detection (bisulfite sequencing) (Chan et al., 2013). Although concentrated targeted regions are advantageous and inexpensive, the former method cannot precisely show the tumor state because methylation regions (especially CpG islands) are easily affected by gene expression levels. The latter method has wide coverage of the sequenced regions, as well as strong prospects for clinical application. At present, there is ample data to support that DNA methylation changes of specific genes in cfDNA, including hypermethylation of TGR5 (Han et al., 2014), MT1M (Ji et al., 2014) and the RASSF1A promoters (Wong et al., 2000), as well as hypomethylation of LINE-1 elements (Tangkijvanich et al., 2007). Not surprisingly, the combinations of cfDNA methylation in panels can improve the diagnostic performance with high sensitivity and specificity of over 90% (Yang et al., 2019). In summary, the methylation panels appear to be a potential detection marker in the clinical utility accompany with promising application prospects.

#### Clinical Applications in HCC

CfDNA was originally discovered in 1948 (Mandel and Metais, 1948). With the recent development of genomics and molecular genetics, cfDNA research has significantly increased for a variety of clinical and research purposes (Bettegowda et al., 2014), and cfDNA analysis is increasingly recognized as an effective and noninvasive tool in the diagnosis, personalized treatment, and follow-up of HCC patients (Table 1).

**TABLE 1:** **|** Clinical Applications of cfDNA in HCC.

Applications	Methods	DNA aberrations	
Detection or screening early HCC			
Integrity	qPCR amplification of b-globin gene	Increased in HBV associated HCC patients.	Chen et al. (2012)
	qPCR	Decreased in HCC patients, increased after hepatectomy in cancer patients and the AUCs for detecting HCC by cfDNA integrity and AFP were 0.705 and 0.605, respectively.	Agostini et al. (2011)
Concentration	real-time PCR amplification	Significantly higher in HCC patients than in HCV carriers without known HCC.	Tokuhisa et al. (2007)
	MiSeq sequencing	There is no difference between HCC patients and healthy people.	Liao et al. (2016)
Mutation	MiSeq sequencing	Genetic mutations were detected in plasma samples of two patients (4.9%) for the TERT genetic mutation, four (9.8%) patients for CTNNB1 mutation, and two (4.9%) patients for the TP53 mutation.	Liao et al. (2016)
	PCR-based sequencing	The median sensitivity of CancerSEEK among the eight cancer types evaluated was 70%, the specificity was 99%.	Cohen et al. (2018)
Methylation	MSRE-qPCR	The combination analysis of these four genes resulted in an increased AUC of 0.933 with 92.7% sensitivity, 81.9% specificity in discriminating HCC from normal control.	Huang et al. (2011)
	the 5 hmC-Seal technique	A 32-gene diagnostic model accurately distinguished early HCC (stage 0/A) from non-HCC (validation set: AUC = 88.4%), showing superior performance over AFP.	Cai et al. (2019a)
guiding treatment			
Mutation	next-generation sequencing	CtDNA derived from noninvasive blood tests can provide exploitable genomic profiles in patients with HCC.	Ikeda et al. (2018)
Prognosis assessment			
Concentration	exome sequencing	Multivariate analysis identified ctDNA (OR 6.10; 95% CI, 1.11–33.33, *p* = 0.038) as an independent predictor of microscopic vascular invasion of the portal vein (VP).	Pezzuto et al. (2018)
Mutation	high-resolution melting PCR (HRM-PCR) and COLD-PCR	Mutated p53 genes could be used as a biomarker of tumor recurrence during the clinical evolution of the transplanted patients.	García-Fernández et al. (2016)
	whole exome sequencing	Real-time track the therapeutic responses in the longitudinal monitoring.	Cai et al. (2017)
Methylation	Targeted bisulfite sequencing	Methylation pattern was highly correlated with tumor burden, treatment response, and stage and can effectively predict prognosis and survival (*p* < 0.001).	Xu et al. (2017)
	real-time quantitative methylation specific PCR (RTQ-MSP)	After surgical resection, the median p16INK4a methylation indices in plasma and buffy coat concordantly decreased 12- and 15-fold.	Wong et al. (2003)
	Methylation Specific PCR (MSP)	Examination of LINE-1 hypomethylation and RASSF1A promoter hypermethylation was effective in predicting early recurrence of HCC after curative resection.	Liu et al. (2017)

CNV, copy number variation; HCC, hepatocellular carcinoma; qPCR, quantitative polymerase chain reactions.

#### Early Detection

The effective management of patients with HCC relies on the early diagnosis of the disease (Lim et al., 2017), while biomarkers for early detection are still deficient. Most recently, improvement of the next-generation sequencing technology and better understanding of genetic or epigenetic alteration of HCC have allowed comprehensive analysis of the landscape of cfDNA. The non-invasive method was also attached great importance in early detection of HCC.

Increasing concentration of cfDNA was observed in the status of illness, thus quantitative measurement of untargeted cfDNA may have diagnostic value for HCC(Tokuhisa et al., 2007). However, due to factors of various assay platforms and physiological state of different patients, there were wide variations in the concentration of cfDNA even among healthy controls. A recent study enrolled 24 HCC patients and 62 hepatitis B virus-related liver fibrosis patients, and constructed a model including age, cfDNA, and AFP, had an area of 0.98 (95% confidence interval 0.92–1.00) under the ROC for the diagnosis of HCC, with 87.0% sensitivity and 100% specificity. Overall, it would not be recommended for using quantitative measurement independently, whereas the combination of cfDNA with other proteins or genetic biomarkers is expected to be a clinical tool for the early diagnosis of HCC (Liao et al., 2016). Compared with the concentration, detecting the cfDNA integrity index is another approach employed in quantitative analysis, which is considered more stable. A study showed that serum DNA integrity in HCC patients was notably higher than that in HBV carriers or healthy controls (Chen et al., 2012). In another study, found that cfDNA integrity was significantly decreased in HCC patients compared with patients with benign diseases and healthy individuals. Additionally, cfDNA integrity (AUC = 0.705) had a higher diagnostic performance than AFP (AUC = 0.605) (Huang et al., 2016a). Considering the heterogeneity of integrity in different studies, it is necessary to comprehensively analyze aberrantly short and long DNA molecules in the plasma of patients.

Cancer-specific genetic aberrations in cfDNA are detectable by liquid biopsy as biomarkers for the diagnosis and monitoring of HCC. As the high heterogeneity in HCC, single gene variation in cfDNA do not have sufficiently high sensitivity and specificity as a biomarker for HCC(Yang et al., 2017; Jiao et al., 2018b; Marchio et al., 2018). For example, a study showed that there were tumor-associated mutations for HCC in only 8 of 41 patient (19.5%) plasma samples, including mutation to such genes as TP53, hTERT, and CTNNB1 (Liao et al., 2016). To achieve an improved ability to diagnose early HCC, different panels of multigene utilizing NGS technology have broader application in the world. A study of 30 biopsy proven HCC patients were prospectively recruited. Using a panel of 46 genes frequently altered in HCCs, deep sequencing of the DNA from the biopsies, cfDNA, and matched germline was performed and ctDNA was detected in 63% of the patients (Labgaa et al., 2018). Joshua D. Cohen et al. developed a method, termed CancerSeek, which combined the 8 serum protein biomarkers with 16 tumor-associated genes in the cfDNA to detect eight types of early (stage I and II) cancers involving 1,005 patients. Among these cancers, CancerSeek has demonstrated accuracy in the diagnosis of early HCC with a sensitivity of 98% and specificity greater than 99% (Cohen et al., 2018). In summary, large panels of targeted NGS or allele-specific assays targeting hotspot mutations are required for early detection, and ctDNA combined with other biomarkers (AFP or other novel circulating molecular biomarkers) may play a crucial supplementary role as a diagnostic biomarker in HCC.

DNA methylation is a core mechanism of epigenetic regulation of gene expression and cell type-, tissue- and organ-specific methylation signature have been exploited as an HCC marker (Villanueva et al., 2015; Lehmann-Werman et al., 2016; Hlady and Robertson, 2018). A single methylation marker candidate study found that the methylation of RASSF1A was detected in 90% of cfDNA in HCC patients. It showed an accuracy rate of 77.5 and 72.5% as a diagnostic marker of HCC in healthy people and HCV carriers, respectively (Zhong et al., 2003). Similar to mutation in cfDNA, combinations of cfDNA methylation in panels can also improve the diagnostic performance in HCC. A model constructed with four methylation genes (APC, GSTP1, RASSF1A, and SFRP1) had a sensitivity of 92.7% and a specificity of 81.9% for detecting HCC(Huang et al., 2011). Furthermore, a recent study developed a 32-gene diagnostic model that accurately distinguished early HCC (BCL 0/A stage) from non-HCC and 15 AFP-negative patients, exhibiting superior performance over AFP (AUC = 88.4%). The model also exhibited superior ability in diagnosing HCC from high-risk populations, such as those with hepatitis and hepatic cirrhosis (AUC = 84.6%) (Cai et al., 2019a). Overall, ctDNA methylation panels appear to have the strongest potential for clinical utility in the early detection of HCC, and all the biomarkers requires prospective validation for the robust performance.

#### Medicine Guidance

Another important application of cfDNA is the identification of tumor gene profiling for individualized treatment, which has become a fundamental practice in cancer medicine (Harding et al., 2019). In the recent decade, various advances in treatment bring great promises and new opportunities for HCC therapeutics. Sorafenib and Lenvatinib in first-line showed efficacy, and regorafenib, cabozantinib as well as ramucirumab in second-line provided more chances for sequential systemic therapy in advanced HCC. Recently, immunotherapy has emerged as one of the most promising approaches to extend current options for needed HCC treatment (Liu et al., 2021a). Frustratingly, less than 40% of HCC patients are eligible for potentially curative therapies. Therefore, it is imperative to detect predictive biomarkers including FGFR4, Cdk5, PD-1, PD-L1, and tumor mutation burden (TMB) which may optimize the therapy strategy and forecast the benefit to patients receiving target and immune therapy. The limited applications of molecular classification using biopsy tissue samples may be attributable intra-tumoral or intertumoral heterogeneity in HCC patients; therefore, one piece of tumor tissue failed to present the complete molecular profile of one HCC patient (Liu et al., 2021b; Liu et al., 2021c). In contrast, numerous studies have demonstrated high concordance rates between plasma and tissue samples, particularly for alterations in key genes (Adalsteinsson et al., 2017; Strickler et al., 2018). Therefore, combined with the features of safety, low cost, operability and dynamic monitoring, cfDNA analysis may possess the potential to provide individualized treatment guidance for HCC patients.

Sadakatsu (Ikeda et al., 2018) implemented NGS to identify actionable mutations in 12 HCC patients, and ultimately, 556 exons of 68 genes were sequenced. A total of 79% of patients harbored at least one actionable mutation alteration, and the median of drug target mutation alterations was 2 (0–5), which suggested that performing liquid biopsies and detecting cfDNA may play a key role in guiding therapeutic decision-making. Furthermore, a patient with a *CDKN2A*-inactivating and a *CTNNB1*-activating mutation received palbociclib (CDK4/6 inhibitor) and celecoxib (*COX-2*/*Wnt* inhibitor); another patient with a PTEN-inactivating and a *MET-ac*tivating mutation received matched treatment: sirolimus (mechanistic target of rapamycin inhibitor) and cabozantinib (MET inhibitor). Eventually, the treatment outcomes of two patients were described as good. Another study prospectively enrolled 121 patients to identify predictors of primary resistance to systematic therapy using cfDNA. Patients with mutations in the PI3K/MTOR pathway had significantly shorter progression-free survival (PFS) than those without these mutations after tyrosine kinase inhibitors (2.1 *vs.* 3.7 months, *p* < 0.001), but not after immune checkpoint inhibition (CPI) (von Felden et al., 2021). Because of the low long-term response to sorafenib in HCC (Méndez-Blanco et al., 2018) and large individual differences in response to immunotherapy (Llovet et al., 2018), few investigations into the use of cfDNA to guide therapy in HCC have been reported.

#### Prognosis Evaluation

Earlier studies showed that the levels of cfDNA in the plasma were significantly correlated with tumor burden (Cai et al., 2017; Pezzuto et al., 2018) with a high specificity and short half-life periods, meaning that cfDNA has a unique strength of prognostic value. There is evidence that mutations in cfDNA are closely related to vascular invasions, and patients with cfDNA mutations are more frequently reported to have shorter recurrence-free survival (RFS) (Cai et al., 2019b). García-Fernández et al. (2016) found that the presentation of TP53 mutations in cfDNA might be used as a biomarker of tumor recurrence in patients with transplanted HCC. Furthermore, Rui-Hua Xu et al. (2017) constructed a prognostic prediction model based on methylation alterations in cfDNA to effectively predict prognosis and survival (*p* < 0.001), which was highly correlated with tumor burden, treatment response, and stage. The above findings demonstrated that changes in cfDNA, including concentration, mutation and methylation, may provide references for the prediction of HCC prognosis.

Benefiting from a short half-life, cfDNA exhibits a fast response to changes in tumor burden after treatment, and thus it is possible to improve prognostication with the detection of tumor-specific molecular alterations. One study reported that the level of cfDNA decreased significantly after surgery, indicating that the dynamic monitoring of cfDNA alterations could provide additional information regarding the presence of residual lesions and early relapse after surgery (Corcoran and Chabner, 2018). ZX found that there was a notable decline in the mutation frequency of cfDNA, and during follow-up, the increased frequency of mutations in cfDNA often revealed the presence of residual lesions and, eventually, relapse (Cai et al., 2017). CtDNA methylation of a panel of genes has shown to be an important prognostic factor. The study of Wong IH showed that RTQ-MSP quantitative analysis can detect epigenetic changes (DNA methylation levels) in peripheral blood from patients with HCC. These researchers observed that the *p16* ^*INK4a*^ sequence of methylation is present in 80% of HCC patients, and a 12-fold decline was observed in the patients after surgery (Wong et al., 2003). Another study indicated that co-evaluation of *LINE-1* hypomethylation and *RASSF1A* promoter hypermethylation was effective in predicting early recurrence of HCC after curative resection (Liu et al., 2017). Furthermore, the level of demethylation was also significantly related to the presence of residual lesions after surgery (Chan et al., 2013). Overall, methylation patterns of the patients after surgery could provide an early warning of residual lesions and early relapse.

#### Challenges and Perspectives

In general, cfDNA may be an informative, inherently specific and highly sensitive biomarker, and can be performed in clinical and research purposes in many types of cancer. A blood sample from peripheral venous blood is not only convenient and easy to implement, but cfDNA in the plasma is relatively stable and easy to extract. In addition, with the characteristic of short half-life, cfDNA in this context is a more accurate and timely reflection of the tumor staging and recurrence than serum biomarkers. With the accumulation of liquid biopsy data, detection panels involving mutations at multiple loci and multiple methylation patterns using cfDNA may be employed for therapeutic monitoring, prognostic evaluation and early diagnosis in HCC.

The descriptions of molecular aberrations of the cfDNA in this review only focus on HCC, while mutations of the same keeper genes, such as TP53, KRAS, and RAF also occur in many other types of cancers. Besides, the cfDNA in plasma mainly derives from rupture and release of blood cells, and the concentration of plasma cfDNA can be affected by the different physiological and pathological conditions. There is an emerging problem which cannot be neglected: how could cfDNA be cancer-specific? The epigenetic biomarkers of cfDNA may be one of the most promising directions. DNA methylation is favored epigenetic biomarker because of cancer-type-specific and tissue-type-specific for the cancer diagnosis and tracing the tissue origin of cfDNA (Gai and Sun, 2019). Moreover, in combination with machine learning, methylation biomarkers in cfDNA provides a new clue to discriminate specific cancer type. Zhou and colleagues modeled the plasma cfDNA as a mixture of DNA derived from tumor and normal tissues, then they used a probabilistic model to sensitively identify a trace amount of tumor cfDNAs in plasma (Li et al., 2018a). Epigenetic biomarkers in cfDNA provide generalizable solutions for early detection and trace the origin of ctDNA.

Although cfDNA performed as a powerful detection and analysis tool along with promising potential, there are still many challenges for this technology to become clinical reality. First, the experimental data described in this review are diverse and vulnerable, which may attribute to a variety of individual differences, experimental designs and detection methods for cfDNA across different studies. In addition, there is a lack of an effective medical therapy and universal actionable mutations for HCC patients, resulting in rare medical applications of cfDNA in the plasma based on HCC-associated gene mutations (Li et al., 2018b). Altogether, cfDNA is an exciting product of precise medicine, and in the near future it may realize the purpose of real-time surveillance and tailored treatment for patients with HCC.

## Abbreviations

AFP, α-fetoprotein; ARMS, amplification-refractory mutation system; AUC, area under the curve; cfDNA, cell-free DNA; CNV, copy number variation; CTCs, circulating tumor cells; ctDNA, circulating tumor DNA; EPIC-Seq, the Epigenetic Profile Inference from the CfDNA Deep Sequencing; HCC, Hepatocellular carcinoma; mmPCR-NGS: massively multiplexed PCR and next-generation sequencing; NGS, next-generation sequencing; qPCR, quantitative real-time PCR; RFS, progression-free survival; ROC, receiver operator characteristic curve; sWGS, shallow WGS; TACE, transplantation or transarterial chemoembolization; TCGA, The Cancer Genome Atlas; UMIs, unique molecule identifiers; WGS, whole-genome sequencing.
